# Non-overlapping functions of Nck1 and Nck2 adaptor proteins in T cell activation

**DOI:** 10.1186/1478-811X-12-21

**Published:** 2014-03-26

**Authors:** Jatuporn Ngoenkam, Pussadee Paensuwan, Kanlaya Preechanukul, Boonruang Khamsri, Ichaya Yiemwattana, Esmeralda Beck-García, Susana Minguet, Wolfgang WA Schamel, Sutatip Pongcharoen

**Affiliations:** 1 Department of Microbiology and Parasitology, Faculty of Medical Science, Naresuan University, Phitsanulok 65000, Thailand; 2 Depatment of Preventive Dentistry, Faculty of Dentistry, Naresuan University, Phitsanulok 65000, Thailand; 3 Department of Molecular Immunology, Faculty of Biology, BIOSS Center for Biological Signalling Studies, Centre for Chronic Immunodeficiency CCI, University of Freiburg, Freiburg, Germany; 4 International Max Planck Research School for Molecular and Cellular biology (IMPRS-MCB), Max Planck-Institute of Immunobiology and Epigenetics, Freiburg, Germany; 5 Centre of Excellence in Medical Biotechnology (CEMB), Faculty of Medical Science, Naresuan University, Phitsanulok 65000, Thailand; 6 Department of Medicine, Faculty of Medicine, Naresuan University, Phitsanulok 65000, Thailand

**Keywords:** Adaptor protein Nck, Nck1, Nck2, shRNA, Jurkat T cells, T cell receptor/TCR activation

## Abstract

**Background:**

Signalling by the T cell antigen receptor (TCR) results in the activation of T lymphocytes. Nck1 and Nck2 are two highly related adaptor proteins downstream of the TCR that each contains three SH3 and one SH2 domains. Their individual functions and the roles of their SH3 domains in human T cells remain mostly unknown.

**Results:**

Using specific shRNA we down-regulated the expression of Nck1 or Nck2 to approximately 10% each in Jurkat T cells. We found that down-regulation of Nck1 impaired TCR-induced phosphorylation of the kinases Erk and MEK, activation of the AP-1 and NFAT transcription factors and subsequently, IL-2 and CD69 expression. In sharp contrast, down-regulation of Nck2 hardly impacts these activation read-outs. Thus, in contrast to Nck2, Nck1 is a positive regulator for TCR-induced stimulation of the Erk pathway. Mutation of the third SH3 domain of Nck1 showed that this domain was required for this activity. Further, TCR-induced NFAT activity was reduced in both Nck1 and Nck2 knock-down cells, showing that both isoforms are involved in NFAT activation. Lastly, we show that neither Nck isoform is upstream of p38 phosphorylation or Ca^2+^influx.

**Conclusions:**

In conclusion, Nck1 and Nck2 have non-redundant roles in human T cell activation in contrast to murine T cells.

## Background

T cells play an important role in adaptive immunity. They express a structurally unique receptor called the T cell antigen receptor (TCR). The TCR is composed of the variable antigen-binding TCRαβ heterodimer noncovalently associated with the non-variable CD3ϵγ, CD3ϵδ and ζζ signal transduction subunits [1]. Binding of the TCR to peptide-loaded major histocompatibility complexes (pMHCs) presented on the surface of antigen presenting cells (APCs) initiates the formation of intracellular protein signalling complexes [2]. The formation of these complexes is required for T cell activation and is driven by adaptor proteins in order to activate downstream pathways, such as the Ras-mitogen-activated protein kinase (MAPK), protein kinase C (PKC) and Ca^2+^-mediated signalling pathways. Stimulation of these pathways results in the activation of the transcription factors activator protein-1 (AP-1), nuclear factor kappa-light-chain-enhancer of activated B cells (NF-κB) and nuclear factor of activated T cells (NFAT), respectively [3]. The cooperative activity of these transcription factors is required for the regulation of interleukin-2 (IL-2) production [4].

Two highly conserved members of the cytosolic adaptor protein Nck (non-catalytic region of tyrosine kinase) are found in humans: Nck1/Nckα and Nck2/Nckβ (also known as Grb4) [5,6]. These two proteins have a 68% similarity when the amino acid sequences are compared [7]. Nck consists of three consecutive Src homology 3 (SH3) domains at N-terminus followed by a SH2 domain at the C-terminus [5]. The SH2 domain of Nck binds to phosphotyrosine-containing proteins, and the SH3 domains bind to proline-rich sequences (PRS) within target proteins [7,8]. In T cells, the SH2 domain of Nck mediates the interaction with phosphorylated SLP-76 (SH2 domain-containing leukocyte protein of 76 kDa), while each of the SH3 domains interacts with distinct target proteins. For instance, SH3.1 of Nck binds directly to a PRS in the cytoplasmic tail of CD3ϵ exposed upon antigen-induced conformational changes within the TCR complex. This interaction is required for IL-2 production and synapse formation [9]. The SH3.2 domain of Nck interacts with the kinase Pak1, which is essential for regulation of the reorganization of actin cytoskeleton [7,10,11] and for activation of NFAT [12]. The SH3.3 domain of Nck binds to WASp to control actin polymerization [13] and with the Son of Sevenless (SOS) [10,14]. The guanine nucleotide exchange factor SOS is required for activation of the Ras-Raf-MEK-Erk pathway [15].

Double Nck1 and Nck2-knockout mice die in early embryonic stage, whereas singly Nck1 or Nck2 deleted mice do not possess any phenotype, suggesting a redundant function of these two molecules in mice [16]. Recently, we have downregulated Nck1 expression in human Jurkat T cells and primary human CD4^+^ T cells using siRNA, showing that Nck1 expression is required for TCR-induced phosphorylation of Erk1/2, IL-2 production and CD69 expression [17]. These results suggest species specific differences in the role of Nck1 and Nck2. The aim of the present study was to compare the roles of Nck1 and Nck2 in TCR-mediated T cell responses in the human system. Our results demonstrate that Nck1 and Nck2 have non redundant roles in human T cell activation. Thus, Nck1 plays a more dominant role in TCR-mediated T cell activation in the human system than Nck2.

## Results

### Generation of Nck1- and Nck2-knockdown Jurkat T cells

**Figure 1:**
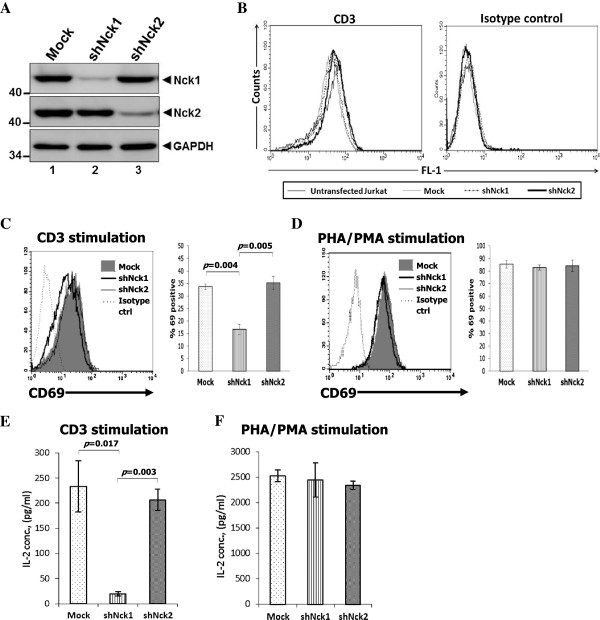
**Nck1 is required for TCR-mediated CD69 expression and interleukin (IL)-2 production. A)** Jurkat T cells were transfected with a control vector, Nck1- or Nck2-specific shRNA expression vector. Transfected cells were selected in medium containing 4 μg/ml puromycin for 4–7 days before cloning by limiting dilution. Cell extracts were subjected to Western blot analysis with anti-Nck1, −Nck2 monoclonal antibody (mAb) and anti-GAPDH mAb. **B)** The expression levels of surface CD3 molecules of the cloned cells from each population were measured by flow cytometry. **C-D)**. Nck1-knockdown (shNck1), Nck2-knockdown (shNck2) and control cells (mock) were stimulated with 1 μg/ml anti-CD3 antibody-coated plates **(C)** or 6 μg/ml PHA plus 1 ng/ml PMA **(D)** for 24 h. Each cell population was stained with anti-CD69 conjugated phycoerythrin (PE) and isotype control antibody and then analysed by flow cytometry. The expression of CD69 was displayed in both histogram (*left*) and bar graph (*right*). For bar graph, the fraction of cells expressing CD69 in each population was normalized to its isotype control. **E-F)** Cells were stimulated as in C-D. The supernatants from CD3 **(E)** and PHA/PMA stimulation **(F)** were measured for IL-2 production by ELISA. Results from CD69 expression and IL-2 production are shown as mean ± SD from triplicate samples and were compared among the groups by using the two-tailed unpaired *t* test. The *p*-value less than 0.05 were considered for statistical significance. Data are representative of three independent experiments.

In this present study, the functions of Nck1 and Nck2 in activation of Jurkat T-cell leukemic line E6-1 were compared. To knockdown either Nck1 or Nck2, Jurkat T cells were transfected with a plasmid encoding either Nck1- or Nck2-specific short hairpin RNA (shRNA) or an empty control plasmid. For generating the stable Nck1- or Nck2-knockdown Jurkat T cell lines, the transfected cells were selected in medium containing puromycin. The resulting bulk population was subcloned by limiting dilution. Individual clones were screened by analyzing Nck1, Nck2 and TCR expression. The selected clones had about 90% downregulation of endogenous Nck1 and Nck2 (Figure 1A) and a similar level of CD3 expression as control cells (Figure 1B). Here, we chose one clone each that will be named shNck1 and shNck2.

### Nck1 is required for TCR-mediated T cell activation

Next, we tested whether the downmodulation of Nck1 or Nck2 had an impact on TCR-induced expression of the early activation marker CD69. Cells were stimulated with anti-CD3ϵ antibodies (OKT3) or with TCR-independent stimuli phytohemagglutinin (PHA)/phorbol myristate acetate (PMA). Nck1-knockdown Jurkat T cells exhibited decreased CD69 expression in response to CD3 stimulation (Figure 1C). In contrast, silencing of Nck2 in Jurkat T cells had no effect on CD69 expression. As a control, a similar level of CD69 expression was observed in Nck1- and Nck2-knockdown cells in response to PHA plus PMA (Figure 1D).

Another important marker for indicating T cell activation is IL-2. Therefore, the production of IL-2 was investigated in Nck1- and Nck2-knockdown cells. Cells were stimulated with anti-CD3ϵ antibodies (OKT3) or TCR-independent stimuli (PHA/PMA). Consistent with our previous report [17], gene-silencing of Nck1 resulted in a significant decrease in IL-2 production (Figure 1E). In contrast, IL-2 production was normal in Jurkat cells transfected with shRNA specific for Nck2 . As expected, no difference in IL-2 production was observed between Nck1- and Nck2-knockdown cells upon stimulation with PHA/PMA (Figure 1F). Taken together, these results showed that in contrast to Nck2, Nck1 was required for TCR-mediated T cell activation in Jurkat T cells.

### Nck1 regulated the activation of Ras-MAPK signalling pathway

Following engagement of TCR, the small GTPases Ras and Rac are activated, and subsequently activate the MAPK signalling cascade. There are three groups of enzymes in the MAPK signalling cascade that are sequentially activated: MAP kinase kinase kinase (MKKK, MEKK or MAP3K), MAP kinase kinase (MKK, MEK, or MAP2K) and MAP kinase (MAPK). Three MAPKs are expressed in T cells: Erk1/2, JNK and p38 [3].

**Figure 2:**
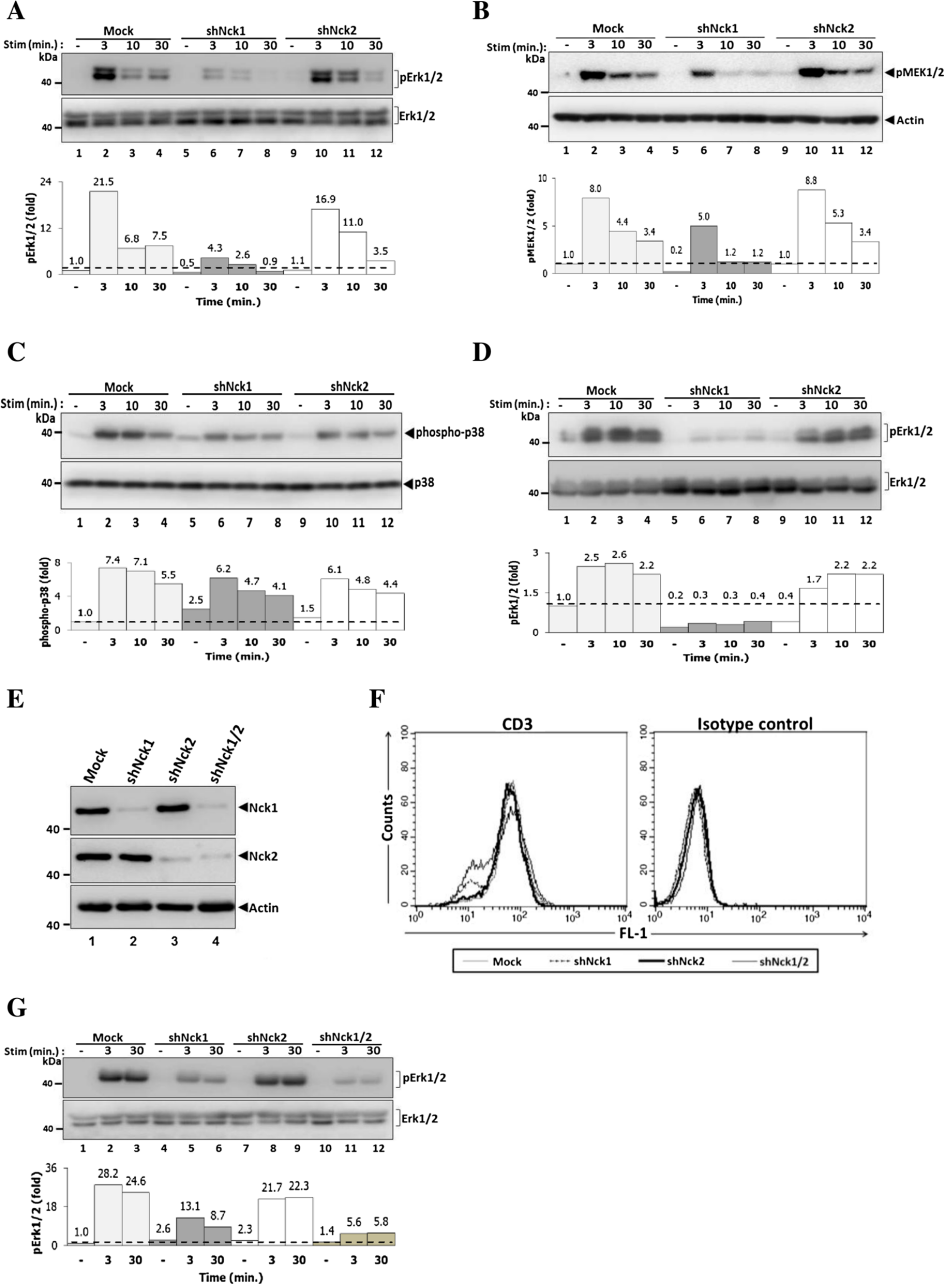
**Nck1, but not Nck2, is essential for activation of Erk1/2 and MEK1/2 proteins.** Cells from each monoclonal group were untreated or treated with soluble CD3 antibody (1 μg/ml) at various time points. Cell lysates were subjected to immunoblot with anti-phospho-Erk1/2 antibody **(A)**. Below, the quantified signal intensity of the pErk1/2 was normalized to its total kinase and this value was relative to that in the unstimulated control cells (Mock), set as 1 (black dashed line) and plotted in bar graph. **B)** Immunoblot analysis of phospho-MEK1/2 from the lysates of cells that were stimulated as in **A**. Below, signal intensity was quantified and presented as a ratio of p-MEK1/2 to actin relative to that in unstimulated control cells (Mock), set as 1 (black dashed line). **C)** Immunoblot analysis of phospho-p38. Below, signal intensity was quantified and presented as described in **A**. **D)**. Polyclonal cells from Mock, Nck1- and Nck2-knockdown cells were stimulated as in **A**. Below, signal intensity was quantified and presented as described in **A**. **E)** Both Nck1 and Nck2 genes were co-silenced in Jurkat T cells. Nck expression levels were analysed by immunoblotting using indicated antibodies. **F)** The expression levels of surface CD3 molecules on double knockdown Nck1/2 were measured by flow cytometry. **G)** Cells from each monoclonal group, Nck1-, Nck2- and Nck1/2-knockdown cells were treated as in A for 0, 3 and 30 min. Lysates were subjected to immunoblot with anti-phospho-Erk1/2 antibody. Below, signal intensity was quantified and presented as described in **A**. Data are representative of at least two independent experiments.

Our previous study has shown that reduced Nck1 expression is associated with impaired Erk1/2 phosphorylation [17]. Nck1 and Nck2 may be differently required for MAPK signalling cascade in Jurkat cells. To test this notion, the activation of two members of MAPK pathway, Erk1/2 and p38 was investigated using Jurkat T cells with silenced expression of Nck1 or Nck2. Reduced Erk1/2 phosphorylation was found in Nck1-, but not in Nck2-knockdown cells (Figure 2A). To confirm that Nck1 is required for activation of the MAPK pathway via the Ras-Raf-MEK-Erk cascade, we investigated the activation of the upstream enzyme essential for Erk1/2 phosphorylation: MEK1/2. As expected, cells treated with shRNA specific for Nck1 exhibited impaired MEK1/2 phosphorylation (Figure 2B). Interestingly, in contrast to Erk1/2 and MEK1/2 phosphorylation, knockdown of Nck1 or Nck2 in Jurkat T cells had no discernible effects on p38 phosphorylation (Figure 2C). Because all these results were obtained from single clones of Nck1- and Nck2-knockdown cells, we confirmed these findings using polyclonal Nck1- and Nck2-knockdown lines to exclude clonal variations. Consistent to the results obtained with single clone cells, the reduction of Erk1/2 phosphorylation was specifically observed in polyclonal Nck1-knockdown, but not in polyclonal Nck2-knockdown lines (Figure 2D). These data show that while Nck1 is required for efficient MEK-Erk activation, Nck2 is dispensable.

To study the possibility of synergistic function of the two Nck isoforms, co-silencing of Nck1 and Nck2 in Jurkat T cells was performed. Jurkat T cells were co-transfected with the Nck1- and the Nck2-specific shRNA expression plasmids, which resulted in downregulation of the expression of both Nck isoforms (Figure 2E). The expression of the TCR on T cell surface of double knockdown cells was similar to each single knockdown population (Figure 2F). Simultaneous knockdown of both Nck1 and Nck2 in Jurkat T cells led to further reduction of TCR-induced Erk1/2 phosphorylation than that seen in Nck1-knockdown cells (Figure 2G). Thus, these data might suggest an additive functions of Nck1 and Nck2 in TCR-induced Erk1/2 activation.

**Figure 3:**
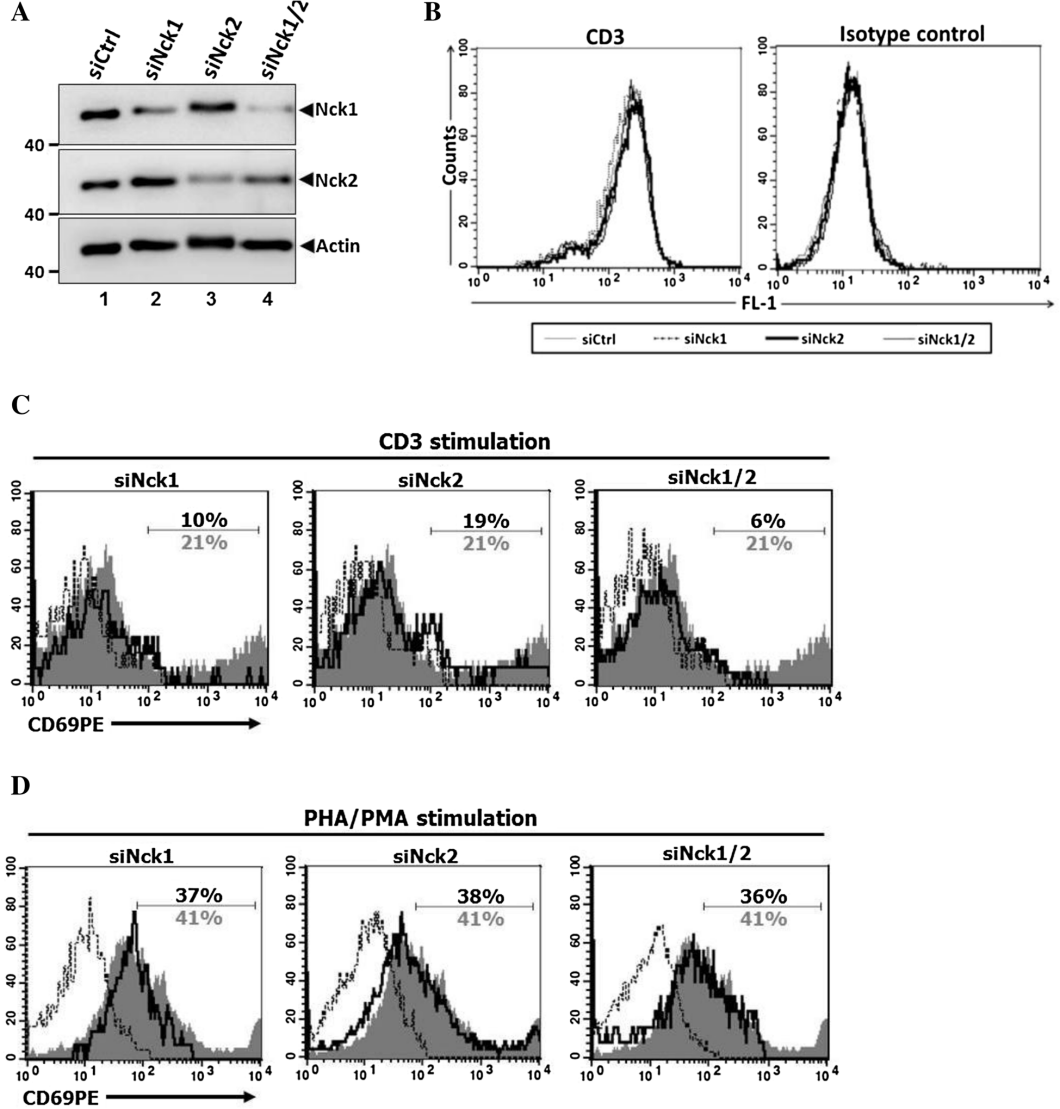
**Nck1 is required for TCR-induced CD69 expression in primary cells.** Peripheral blood mononuclear cells (PBMC) were isolated from buffy coat derived from healthy blood donors. Cells were treated with siRNA specific to either Nck1 or Nck2 and treated with both siRNA specific to Nck1 and Nck2. **A)** The expression of Nck was analysed by immunoblotting. **B)** The expression levels of surface CD3 molecules on these knockdown primary cells were measured by flow cytometry. **C-D)** siRNA-treated cells were stimulated either with anti-CD3 antibody or PHA/PMA for 24 hr. Cells were then harvested and stained with PerCP-conjugated mouse anti-human CD4 mAb and PE-conjugated mouse anti-human CD69 antibody before analysing by flow cytometry. Numbers in CD69 histogram indicate frequency of positive cells within a CD4 gated population. Grey shaded histrogram and grey letter are cells treated with control siRNA, black bold solid line and black letter are cells treated either with siRNA specific to Nck1 or Nck2, and black dotted line is isotype control staining.

To confirm that the results observed in human leukemic T cell lines were also reproduced in primary cells, human PBMCs were used to study the function of Nck1 and Nck2 in T cell activation. PBMCs were transiently transfected with siRNA specific to either Nck1 or Nck2 or both Nck1 and Nck2. In comparison with cells treated with control siRNA, cells treated with specific siRNA showed only reduction in the expression for the corresponding Nck isoform (Figure 3A) without affecting the surface TCR levels (Figure 3B). Consistent with Jurkat T cells, PBMCs treated with siRNA specific to Nck1, but not Nck2, exhibited an impairment of TCR-induced surface expression of CD69 (Figure 3C). Interestingly, inhibition of both Nck1 and Nck2 in PBMCs led to an additive defect in CD69 expression following CD3 stimulation. However, silencing of either Nck1 or Nck2 and co-silencing of Nck1 and Nck2 simultaneously had no effect on CD69 expression in response to TCR-independent stimulation using PHA/PMA (Figure 3D). These data confirmed the importance of Nck1 in TCR-mediated T cell activation also in human primary cells and point out a possible collaboration between Nck1 and Nck2 to optimally up-regulate CD69 upon TCR-activation.

### Nck1 and Nck2 are not required for Ca^2+^ mobilization

**Figure 4:**
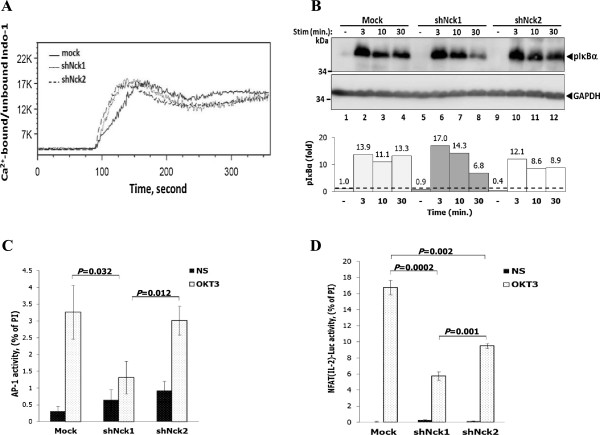
**Dispensable role of Nck protein in TCR-induced Ca**^**2+ **^**mobilization. A)** Cells were loaded with Indo-1 AM and baseline was measured for 1 min before stimulation with OKT3 antibody. The ratio of Ca^2+^-bound and -unbound forms of Indo-1 AM was monitored by flow cytometry. Data are representative from three independent experiments. **B)** Cells were stimulated with soluble anti-CD3 antibody (1 μg/ml) at various time points. Lysates were immunoblotted with anti-phospho-IκBα (Ser32) antibody. Below, signal intensity was quantified and presented as a ratio of p-IκBα to GAPDH relative to that in unstimulated control cells (Mock), set as 1 (black dashed line). Data are representative of two independent experiments. **C)** Cells were transiently co-transfected with pAP-1-Luc and control pGL4.7 plasmid. After 20 hr of transfection, cells were left untreated or stimulated with plate-bound monoclonal antibody to CD3, or with a combination of PMA and ionomycin. Cells were then lysed and measured for the luciferase activity. To control for the transfection efficiency, the pAP-1-Luc activity was normalized to the *Renilla* luciferase activity. Bars represent the mean luciferase activities ± SD from triplicate wells and expressed as percentage of the response to PMA plus ionomycin (PI) and are representative of two independent experiments. **D)** Each cell population was transiently co-transfected with the pNFAT(IL2)-Luc reporter plasmid plus control pGL4.7 plasmid. After 20 hr of transfection, cells were carried out as described above. Bars represent the mean luciferase activities ± SD and expressed as percentage of the response to PMA plus ionomycin (PI). The results were compared among the groups by using the two-tailed unpaired *t* test. The *p*-value less than 0.05 were considered for statistical significance. The data are representative of two independent experiments.

In addition to MAPK signalling pathway, the involvement of Nck1 and Nck2 in other pathways was assessed. Nck has been suggested to be implicated in Ca^2+^ signalling in the mouse system, since Nck-knockout mice showed impairment of Ca^2+^ mobilization in response to TCR stimulation [18]. Thus, whether human Nck1 and Nck2 are involved in Ca^2+^ immobilization was investigated. In contrast to the mouse system, knockdown of Nck1 or Nck2 in Jurkat T cells did not perturb Ca^2+^ influx following TCR stimulation at optimal anti-CD3 antibody concentration (Figure 4A). Moreover, no differences in Ca^2+^ influx were observed between Nck1- or Nck2 knockdown cells following stimulation at limiting concentration of the stimulating antibody (data not shown). Thus, in Jurkat T cells, Ca^2+^ mobilization is independent of Nck1 or Nck2 expression upon TCR triggering.

Next, we investigated IκBα phosphorylation upon TCR triggering. IκBα is phosphorylated downstream of protein kinase C (PKC). It was found that downregulation of neither Nck1 nor Nck2 altered IκBα phosphorylation in response to TCR stimulation (Figure 4B). Altogether, these data indicated that Nck1 or Nck2 are dispensable for Ca^2+^ mobilization and IκBα phosphorylation in Jurkat T cells.

IL-2 gene transcription requires NFAT-AP-1 complexes binding to the *IL*-*2* promoter region. Due to the impairment of IL-2 secretion in Nck1-knockdown Jurkat T cells, the activation of transcription factors AP-1 and NFAT was investigated. Nck1- and Nck2-knockdown Jurkat T cells were transfected with luciferase reporter plasmids containing either an AP-1 binding site or three tandem repeats of the distal NFAT biding sites of the IL-2 gene promoter (NFAT (IL2)). In contrast to Nck2- knockdown cells, Nck1-knockdown cells showed significantly decreased TCR-induced AP-1-dependent luciferase expression (Figure 4C) as compared to control cells. However, TCR-induced NFAT (IL2) activation was statistically impaired in both Nck1- and Nck2-knockdown cells when compared with control cells (Figure 4D). Although Nck2-knockdown cells had a defective NFAT activation when compared to control cells, they retained the ability to maintain TCR-mediated IL-2 production to normal levels (Figure 2C). These results, at least in part, suggest that Nck1 contributed to AP-1 and NFAT (IL2) activation and their simultaneous impairments eventually abrogated IL-2 production.

### The C-terminal SH3 domain of Nck1 controls activation of the Erk1/2 pathway and CD69 expression

**Figure 5:**
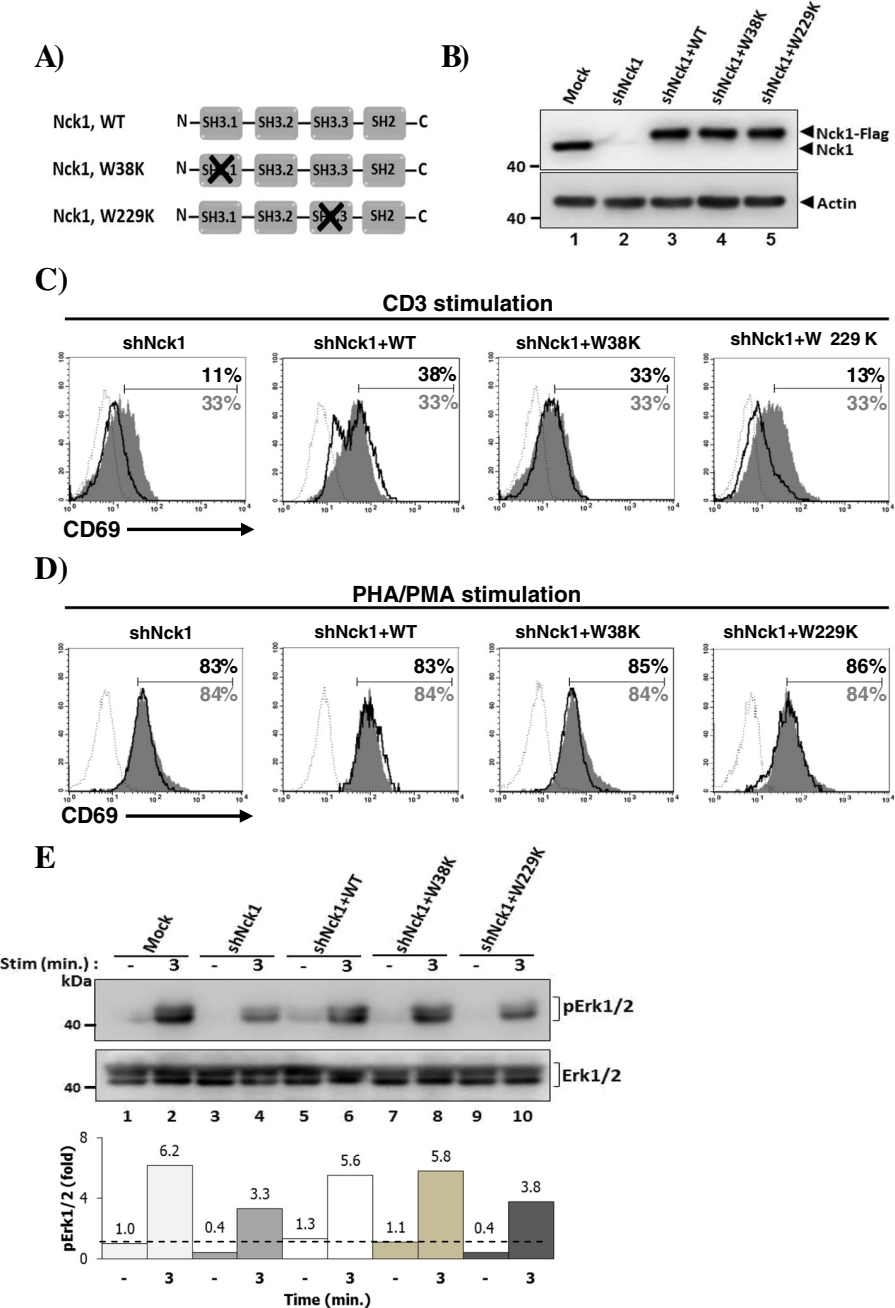
**The C-terminal SH3 domain of Nck1 is necessary for an efficient Erk1/2 activation. A)** Schematic presentation of Nck1 SH3.1 and SH3.3 mutant constructs. A nucleotide fragment encoding the FLAG peptide was linked in frame to the N-terminal of the Nck1 gene. N-terminal SH3 (SH3.1) and C-terminal SH3 (SH3.3) domains of Nck1 were mutated by changing tryptophan (W) residue at 38 and 229 to lysine (K), respectively. **B)** The reconstitution of WT Nck1, Nck1 W38K and Nck1 W229K in Nck1-knockdown cells were analyzed by immunoblotting with anti-Nck1 antibody. **C-D)** Nck1-knockdown cells stably expressing WT Nck1, Nck1 W38K and Nck1 W229K mutants were stimulated with plate pre-coated with 1 μg/ml anti-CD3 antibodies or 6 μg/ml PHA plus 1 ng/ml PMA for 24 h. Each cell population was stained with anti-CD69 conjugated phycoerythrin (PE) and isotype control antibody and then analysed by flow cytometry. Numbers in CD69 histogram indicate frequency of positive cells. Grey shaded histrogram and grey letter are cells transfected with empty plasmid (Mock), black bold solid line and black letter are Nck1-knockdown cells or Nck1-knockdown cells reconstituted with indicated construct plasmids, and black dotted line is isotype control staining. Data are representative of two independent experiments. **E)** Nck1-knockdown cells reconstituted as describe in C were left untreated or treated with soluble CD3 antibody (1 μg/ml) for 3 min. Lysates were immunoblotted with anti-phospho-Erk1/2 (Thr202/Tyr204, Thr185/Tyr187) antibody and anti-Erk1/2 antibody. Below, the quantified signal intensity of the pErk1/2 was normalized to its total kinase and this value was relative to that in the unstimulated control cells (Mock), set as 1 (black dashed line) and plotted in bar graph. Data are representative of two independent experiments.

In human myelogenous leukemia cell line, the C-terminal SH3 (SH3.3) domain of Nck has been documented to bind to SOS, a guanine nucleotide exchange factor for Ras. It was also suggested that other SH3 domains of Nck1 might be implicated in high affinity binding to SOS [14]. An interaction of Nck to SOS implies that Nck is involved in Ras activation, which stimulates various downstream signalling proteins including Erk1/2. In this present study, we performed point mutation at either SH3.1 or SH3.3 domain of Nck1 by changing the tryptophan residue at position 38 or 229 within the conserved WW motifs to lysine corresponding to SH3.1 and SH3.3, respectively [19] (Figure 5A). This residue has been reported as the essential site for binding to its partner without affecting the binding activity of the unmutated domains [20]. The protein expression of reconstituted plasmids encoding wild type (WT) Nck1 and Nck1 mutants tagged with Flag was monitored by immunoblotting (Figure 5B).

The impaired CD69 expression induced by CD3 stimulation was specifically restored in Nck1-knockdown cells reconstituted with WT Nck1 and Nck1 W38K, but not with Nck1 W229K (Figure 5C). Importantly, the reconstitution of Nck1 W229K domain to Nck1-knockdown cells caused CD69 upregulation in response to TCR-independent stimulation (PHA/PMA) equivalent to that in cells reconstituted WT Nck1 and Nck1 W38K (Figure 5D). Furthermore, in contrast to mutation at Nck1 SH3.1, mutation at Nck1 SH3.3 domain did not restore TCR-induced Erk1/2 phosphorylation (Figure 5E). Altogether, the SH3.3 domain of Nck1 plays an important role in regulation of TCR-mediated T cell activation.

## Discussion

Our previous report has shown that Nck1 is required for Jurkat T cell activation [17]. In the present study, we characterize the roles of Nck1 and Nck2 in human T cell activation using Jurkat T cells as a model and CD4+ primary T cells. Our results show that knockdown of Nck1, but not Nck2, caused an impaired IL-2 production and CD69 expression. These impairments were accompanied by the reduction of Erk1/2 and MEK1/2 phosphorylation, which were observed in Nck1-knockdown T cells, but not in Nck2-knockdown T cells. In contrast to MEK-Erk activation, neither downregulation of Nck1 nor Nck2 had any discernible effects on p38 phosphorylation. Although Erk1/2 and p38 are MAPK family members, their activation differentially relies on Ras and Rac, respectively [3,21]. Notably, Ras activation is controlled by SOS [15] and it has been found that Nck can interact with SOS [10,14]. This interaction is mediated predominantly by SH3.3 domain of Nck and proline-rich region of SOS. Although the single SH3.3 domain of Nck mediates this interaction, the high affinity binding of Nck to SOS requires other SH3 domains of Nck. Since the activation of Ras is essential for Erk1/2 activation, CD69 expression and IL-2 gene transcription [22], we performed a point mutation in the SH3.1 (Nck1 W38K) and SH3.3 (Nck1 W229K) domains of Nck1. These mutated genes were then expressed in Jurkat cells in which Nck1 was previously silenced. In contrast to Nck1 W38K, our results clearly show that the Nck1 W229K did not restore the function of full length Nck1 in respect to CD69 upregulation or Erk1/2 phosphorylation upon TCR triggering. Thus, we highlighted the important roles of the SH3.3 domain of Nck1 in linking TCR triggering and Ras-ERK pathway activation in Jurkat T cells.

The cooperative binding of AP-1 and NFAT to promoter/enhancer regions of IL-2 gene is absolutely required to elicit IL-2 transcription [23,24]. Using luciferase reporter assays, we have detected a specific reduction of AP1-mediated transcription in Jurkat cells in which Nck1 was silenced. In contrast, Nck2-silenced cells show transcription levels comparable to control Jurkat cells. The reduction in AP1-mediated transcription in Nck1-knockdown cells is probably related to the reduction in TCR-induced Erk phosphorylation, whereas these events were normal in Nck2-knockdown cells. Reduced AP1-transcriptional activity as consequence of impaired Erk activation has been previously reported [25,26].

Interestingly, although the TCR-triggered Ca^2+^ influx was not altered in Nck1- and Nck2-knockdown T cells, these cells failed to induce downstream NFAT (IL2)-dependent transcriptional responses. One possible explanation is that both Nck isoforms are involved in the transduction of the activation signal downstream of Ca^2+^ influx. Another possibility might be that there is an alternative pathway, apart from Ca^2+^ signalling, that links TCR triggering to NFAT activation. Consistent with this hypothesis, it has been reported that following TCR stimulation, Pak1 is activated and associates with the SH3.2 domain of Nck [12]. Pak1 can be activated independent of Ras activation or calcium flux and this occurs downstream of Vav and Cdc42. The association of Nck-Pak1 is essential for TCR-mediated activation of NFAT transcription factor, since inhibition of NFAT transcriptional activity has been observed when the SH3.2 domain of Nck was mutated such that it failed to bind to Pak1. Although the mechanisms by which both Nck isoforms play non-redundant role in TCR-induced NFAT activation are not known, AP-1 activity was not reduced and NFAT activity was less reduced in Nck2-knockdown cells as compared with Nck1-knockdown cells. Therefore it might be that together the transcription was over the threshold needed to exhibit normal IL-2 production.

Reduction of NFAT in Nck2-knockdown cells did not affect the levels of IL-2 production. AP-1 activation was unaltered in Nck2-knockdown cells. It is possible, that low amounts of active NFAT may be sufficient to form a complex with normal amounts of AP-1 to induce IL-2 production. In contrast, in Nck1-knockdown cells AP-1 and NFAT activities are reduced such that they cannot optimally promote IL-2 transcription. Indeed, it has been previously shown that NFAT activation in the absence of AP-1 activation substantially decreases the IL-2 production [24].

Nck1 and Nck2 protein sequences share only 68% amino acid identity both in humans and mice. However, mouse Nck1 is compared with human Nck1 and mouse Nck2 with human Nck2, the amino acid identity is over 95% [27]. Mice with single knock-out of Nck1 or Nck2 do not have any apparent phenotype whereas double knock-out mice die in the early embryonic stage. Thus, a functional redundancy between these Nck isoforms in mice was proposed [16]. Although, there are evidences that Nck1 and Nck2 share a redundant role in TCR-induced actin polymerization in Jurkat T cells [28], this present report identified the non-overlapping function of Nck1 and Nck2 in TCR signalling and activation in the human system. Thus, it seems that Nck1 and Nck2 have redundant roles in the mice system but not in the human system. One explanation could be the 5% of non-identical sequence or species-specific interacting partners. However, an alternative explanation could be that in the knock-out mice the cells have evolved with only one of the Nck isoforms, and thus they have adapted their signalling pathways to efficiently function under these conditions. In contrast, our approach leads to an abrupt decrease in the expression of one of the isoforms, the plasticity of the mature cell is thus not enough to adapt to the new conditions.

## Conclusions

In conclusion, our results demonstrate that two closely related adaptor proteins Nck1 and Nck2 are functionally non-redundant in TCR-mediated T cell activation. Nck1 plays a major role than Nck2 when controlling TCR-induced transcription and protein expression. In addition, the third SH3 domain of Nck1 is critical for TCR-mediated T cell activation.

## Methods

### Antibodies

Mouse anti-CD3 (OKT-3) antibody was purchased from e-Bioscience (eBioscience, San Diego, CA, USA). Anti-Nck1, anti-phospho-MEK1/2 and anti-phospho-p38, antibodies were from Cell Signaling (Cell Signaling Technology, Danver, MA, USA). Anti-Nck2 antibody was purchased from Abnova (Abnova Corp., Taipei, Taiwan). Anti-phospho-Erk1/2 antibody was from Upstate (Upstate Biotechnology, Lake Placid, NY, USA). Anti-phospho IκBα antibody was supplied from Santa Cruz (Santa Cruz Biotechnology, Santa Cruz, CA, USA). FITC-conjugated anti-CD3 and PE-conjugated anti-CD69 antibodies were purchased from eBioscience (eBioscience, San Diego, CA, USA).

### Construction of Nck1- and Nck2- specific shRNA and transfection

The Nck1- and Nck2-specific RNA interference (RNAi) sequences that were used to generate Nck1 and Nck2 shRNA have been described previously [17,28]. Upper and lower strand of shRNA oligonucleotides specific to each Nck isoform were purchased from Invitrogen (Invitrogen, Carlsbad, CA, USA). The sequence of 5′- to 3′-end oligonucleotides were GATCCGGGGTTCTCTGTCAGAGAAATTCAAGAGA TTTCTCTGACAGAGAA CCCTTTTTTACGCGTG for shNck1 upper strand, AAT TCACGCGTAAAAAAGGGTTCTCTGTCAGAGAAATCTCTTGAATTTCTCTG ACAGAGAACCCCG for shNck1 lower strand, GATCC GCTTAAAGCGTCAGGG AAGATTCAAGAGATC TTCCC TGACGCTTTAAGTTTTTTACGCGTG for shNck2 upper strand, and AATTCACGCGTAAAAAACTTAAAGCGTCAGGGAA GATCTCTTGAATCTTCCC TGACGCTTTAAGCG for shNck2 lower strand. Each counterpart oligonucleotides were annealed and then inserted into pLVX-shRNA1 vector at *Bam*HI and *Eco*RI sites (Takara Shuzo, Tokyo, Japan). The resulting vectors were verified by DNA sequencing before transfection into Jurkat T cells.

### Cell culture and transfection

In order to knockdown either Nck1 or Nck2 protein in Jurkat T cells, cells were cultured and transfected as previously described [17]. Briefly, 2.0 × 10^5^ cells were transfected either with 0.5 μg of pLVX-shNck1 or pLVX-shNck2 or both plasmids. Transfected cells were then incubated at 37°C in a humidified CO_2_ incubator for 48 hr. Stable Nck1- and Nck2-knockdown Jurkat T cell lines were obtained by culturing transfected cells in medium containing 1.0 μg/ml puromycin for 4–7 days. Then, the Jurkat T cell clones were obtained from these lines by limiting dilution technique. Clones that provided a similar level of TCR surface expression as evaluated by flow cytometry were selected for further studies.

Peripheral blood mononuclear cells (PBMC) were isolated from buffy coats obtained from normal blood donors at the Blood Bank Center of Naresuan University Hospital. The used of buffy coats was approved by the ethical committee of Naresuan University. The PBMCs were transfected as previously described [17]. Human Nck1 and Nck2 small interfering RNA (siRNA) oligonucleotides were purchased from Invitrogen. The transfected PBMCs were subsequently cultured in RPMI-1640 plus FBS for 48 h before performing functional study.

### Flow cytometry

The expression of CD3 molecules on T cell surface were determined using anti-CD3 conjugated FITC antibody. To determine the expression of CD69, Jurkat and primary cells were stimulated as previously described [17] and stimulated cells were stained with anti-CD69 conjugated phycoerythrin (PE) antibody. Stained cells were analyzed on a FACSCalibur (Becton Dickinson, NJ, USA) and data were analyzed with CellQuestPro software.

### Analysis of IL-2 production

The measurement of IL-2 production was performed as previously described [17]. Briefly, cells at 1 × 10^6^ cells/ml were incubated in micro titer plates that were pre-coated with 1 μg/ml of anti-CD3ϵ monoclonal antibody (mAb) or with 6 μg/ml of phytohaemagglutinin (PHA) and 1 ng/ml of phorbolmyristate acetate (PMA) for 24 hours at 37°C. The supernatants were collected and stored at −80°C until assayed. IL-2 levels were determined using a commercial enzyme-linked immunosorbent assay (ELISA) kit (R&D, Minneapolis, MN, USA) following the manufacturer’s instructions. The optical density at 450 nm was read using a microplate reader (Perkin Elmer, MA, USA).

### Western blotting analysis

A total of 5×10^6^ cells was resuspended in serum-free RPMI medium and incubated in an incubator for 60 min. Cells were left untreated or treated with anti-CD3 (OKT3) antibody at different times points. After incubation at each time point, cells were lysed in Brij96 lysis buffer containing protease and phosphatase inhibitors (20 mM Tris–HCl (pH 8.0), 137 mM NaCl, 2 mM ethylenediamine tetraacetic acid (EDTA), 10% glycerol, 10 mg/ml leupeptin, 10 mg/ml aprotinin, 1 mM phenylmethylsulphonyl fluoride (PMSF), 500 mM sodium orthovanadate, 1 mM sodium fluoride (NaF) and 0.5% Brij96). The samples were left on ice for 15 min and then centrifuged at 12,000 rpm for 15 min. The supernatants were collected and 25 μg of total protein were loaded in each lane of SDS-PAGE and western blotting analysis according to standard procedures. The membranes were probed with anti-phospho-specific antibodies and observed under a CCD camera (ImageQuant LAS 4000; GE Healthcare Life Sciences, Pittsburgh, PA, USA). Relative band intensity was quantified by ImageJ software and shown as the mean ± SE.

### Ca^2+^ influx measurement

Five million cells were resuspended in 1 ml RPMI supplemented with 1% fetal calf serum (FCS) and incubated with calcium indicator Indo-1 AM (2 μM; Molecular Probes, Invitrogen) for 45 min at 37°C in 5% CO_2_ with gentle vortexing every 15 min. Cells were washed twice and finally resuspended with 2 ml RPMI supplemented with 1% FCS. Cells were pre-warmed to 37°C before analysis and were maintained at 37°C during event collection on a Becton Dickinson (BD) LSR II Flow Cytometer (Beckton Dickinson). Baseline fluorescence was monitored for 1 min. After 1 min data acquisition began, anti-CD3 antibody (OKT3) at a final concentration of 1 μg/ml was added and the stimulation was induced for 5 min. Kinetic analysis was performed with FlowJo kinetics platform (FlowJo v8.8.4 software (Tree Star)) and data were presented as the ratio of Ca^2+^-bound and unbound Indo-1.

### Luciferase assay

The transfection procedure to introduce the luciferase reporter plasmid into the cells was performed. 2×10^5^ cells were transiently co-transfected with 0.45 μg of a firefly luciferase reporter plasmid containing either AP-1 (pAP-1-Luc) or NF-AT binding sites (pGL3 NFAT-Luc) and 0.05 μg pGL4.7 control plasmid (*Renilla* luciferase plasmid). After 16–20 hr, cells were harvested and 1×10^5^ cells were cultured at 37°C for 6 h in complete medium alone (unstimulated), or stimulated either with plate-bound anti-CD3 mAb, or with 50 ng/ml phorbol 12-myristate 13-acetate (PMA) and 1 mM ionomycin. After stimulation period, cells were lysed and assayed for luciferase activity according to the manufacturer’s instructions. To correct the variations in transfection efficiency, the values of NFAT luciferase activity were normalized with the activity of the *Renilla* luciferase.

### Generation of Nck1 mutant and transfection

All mutants were generated by site-directed mutagenesis of double-stranded DNA using QuickChange® Lightning Site-Directed Mutagenesis kit (Stratagene, La Jolla, CA). Human FLAG-tagged Nck-1 cDNA containing in pEBB plasmid was kindly provided by Prof. Bruce Mayer, the Connecticut University Health Center, Farmington, CT, USA. Nck1 wild type (WT) was rendered resistant to Nck1 shRNA by site-directed mutagenesis with primers indicated below. The Nck1 mutant was done on the SH3 domain by changing the first tryptophan (W) residue in the conserved WW motif to lysine (K) [19]. The primers for doing these mutations were: Nck1 resistance forward 5′-GTGACCATGTGGGTTCCCTCTCCGAGAAATTAGC AGC-3′, Nck1 resistance reverse 5′-GCTGCTAATTTCTCGGA GAGGGAACCCA CATGGTCAC-3′, Nck1 (W38K) forward 5′-GCTTCTGGATGATTCTAAGTCCAA GTGGCGAGTTCGAAATTCC-3′ and Nck1 (W38K) reverse 5′-GGAATTTCGAAC TCGCCACTTGGACTTAGAATCATCCAGAAG C-3′, Nck1 (W229K) forward 5′-CCTGAAAATGA CCCAGAGAAGTGGAAATGCAGGAAGATCAATGG-3′and Nck1 (W229K) reverse 5′-CCATTGATCTTCCTGCATTTCCACTTCTCTGGGT CATTTTCAGG-3′.

PCR reactions and conditions were performed according to manufacturer’s instruction (Stratagene). The resulting DNA was then confirmed by DNA sequencing analysis. To generate Nck1-knockdown Jurkat cells stably expressing wild type Nck1, or the Nck1 mutant, a total of 2 × 10^5^ cells were co-transfected with pEBB plasmid containing either WT Nck1, Nck1 W38K, or Nck1 W229K (0.4 μg DNA) and pDsRed-Monomer-Hyg-N1 (0.1 μg DNA) (Clonetech, Mountain View, CA, USA), which provides resistance to the antibiotic hygromycin. Fourty-eight hours following transfection, transfected cells were selected for 3–5 days in media containing 400 μg/ml hygromycin. Selected cells were screened for Nck1 re-expression by immunoblotting.

### Statistical analysis

Statistical analyses were performed using SPSS software. All differences between experimental groups were analysed with student’s t-test. The differences were considered to be significant when *P* <0 · 05.

## Competing interests

The authors declare that they have no competing financial and non-financial interests.

## Authors’ contributions

JN carried out laboratory work including plasmid construction, biochemical studies, ELISA, flow cytometry and statistical analyses. PP did PBMCs isolation and construction of Nck1-shRNA plasmid. KP, BK and IY participated in planning the study. WWS, SM, EB helped in planning the biochemical studies. SP and JN planned the study and drafted the manuscript. All authors read and approved the final manuscript.
